# Technological parameter optimization for walnut shell-kernel winnowing device based on neural network

**DOI:** 10.3389/fbioe.2023.1107836

**Published:** 2023-02-02

**Authors:** Hao Li, Yurong Tang, Hong Zhang, Yang Liu, Yongcheng Zhang, Hao Niu

**Affiliations:** ^1^ College of Mechanical Electrification Engineering, Tarim University, Alar, China; ^2^ Agricultural Engineering Key Laboratory, Ministry of Higher Education of Xinjiang Uygur Autonomous Region, Tarim University, Alar, China

**Keywords:** neural network, winnowing device, CFD-DEM, walnut, technological parameter optimization

## Abstract

The detection method for technological parameter is outdates as the traditional test cycle is long as well as the measurement error and the test amount are huge. Moreover, it is difficult to disclose the operation mechanism of devices as the operation is time-consuming and laborious. Therefore, numerical simulation was used in this study to reveal the mechanism of the walnut shell-kernel winnowing device. Moreover, the influence of baffle opening combinations, inlet wind velocity and inlet angle on cleaning rate and loss rate was predicted by the neural network model. The results demonstrated that inlet wind velocity was the primary influencing factor of cleaning rate, followed by baffle opening and inlet angle. Besides, inlet wind velocity was the primary influencing factor of loss rate, followed by inlet angle and baffle opening. The winnowing device performed best (79.91% cleaning rate, 14.37% loss rate) when the baffle opening, inlet wind velocity and inlet angle were 7.01 cm, 24.36 m/s, and 9.47°. In addition, 1/8 walnut shells and 1/4 walnut kernels were incorrectly classified due to the increase in inlet wind velocity. The inlet wind velocity was considered the major cause behind the deteriorating winnowing performance of the device. Finally, the bench test and simulation optimization results were compared. The cleaning rate and loss rate relative error during the simulation test was lower than 1.06%, which ascertained the feasibility and validity of the neural network as well as the combined numerical simulation method. This study could be useful for future research and development of shell-kernel winnowing devices for hard nuts.

## 1 Introduction

Post-harvest processing of walnut can improve the economic added value. Shell breaking and kernel collection are crucial steps during the post-harvest process, out of which shell-kernel separation is the primary step (Liu et al., 2021). Common shell-kernel separation methods include winnowing (Nahal et al., 2013), image (Jiang et al., 2007), magnetic selection (Krishnan and Berlage, 1984) and floating selection (Romberg, 1938). Among them, the winnowing method is mostly used due to its simple principle and low cost. The winnowing effect could be influenced by real-world engineering challenges by a number of factors including material state, structural parameters and device operation parameters (Liu et al., 2021). Therefore, understanding the winnowing mechanism of the device and the optimal parameters is crucial.

The selection of winnowing is usually differentiated based on the difference between the suspension speed of each material. Airflow thrust acts on the material during the winnowing selection process. Therefore, the material moves along the direction of the joint force while completing the screening operation. In the actual experiments, the size and direction of airflow thrust are usually controlled by adjusting inlet wind velocity and inlet angle. The windward condition of materials is typically controlled by adjusting the baffle opening. The traditional test methods could face dual constraints of periods and costs during the exploration of the operation mechanism of devices and could encounter multiple limitations in information acquisition (Golshan et al., 2021). Numerical simulation methods can study the phenomenon production process and operation mechanism from the microscopic perspective while saving time and labor costs compared to traditional tests (Qi et al., 2015; Lingfeng et al., 2019). To understand the drag reduction principle of subsoiling mechanical bionic structure, Lingfeng et al. (2019) established a soil model through the Discrete element method (DEM). It simulated the action relationship between the bionic structure and soil particles under different cultivation conditions and thoroughly investigated the drag reduction principle of the bionic structure. Akrami et al. (2021) simulated the natural ventilation system of a greenhouse by using computational fluid dynamics (CFD) technology, which disclosed the action principle of inlet position on the ventilation system by analyzing the influence of different ventilation openings on ventilation performance. Chen et al. (2022) simulated a particle collision process in the separation tube through CFD-DEM coupling to improve the separation performances of the brown rice screening device. They determined the loss of brown rice as well as the wrong screening mechanism of rice husks. The numerical simulation can provide detailed practical data which could be useful to understand the device operation mechanism. However, the drastic increase in computational resource needs for numerical simulation during large-scaled optimization tests cannot be ignored. Fortunately, researchers have found that the alternative model based on artificial neural networks can be used as a reducing model to effectively reduce the demand for computing resources. For example, Kolarik and Rudorfer (1994) have demonstrated that the prediction ability of artificial neural networks was better than the self-regression model. At the same time, Michael Thomas Rex et al. (2020) established the response surface method and artificial neural network prediction model by using the findings of finite element analysis and demonstrated the ability of the artificial neural network in modelling optimization. In order to address the poor fitting degree and low accuracy in multi-objective parameter optimization, Dong et al. (2022) used a straw-returning device as the research object and obtained the optimal parameter combination of associated experimental factors through a neural network optimization model. Therefore, it is essential to apply neural networks having strong prediction ability and fast optimization capability for the optimization of parameters. To improve the tractive performances of tractors under low fuel consumption and a low degree of soil compaction, Pentoś et al. (2020) introduced a high-precision mathematical model. They utilized the neural network and indicated soil type as the major influencing factor of tractive performances. To investigate the effect of temperature, wind speed and time on the drying of *Artemisia absinthium* leaves, Karimi et al. (2012) constructed a prediction model by combining neural network and response surface methodology. They completed parameter optimization during the *A. absinthium* leaves drying process and improved the drying effect. In order to understand the macro-mechanical characteristics of granular materials, Dosta and Chan (2022) used bonded particles model (BPM) for a single-axis compression test of the cluster. At the same time, the artificial neural network model with aggregates as input parameters was established. Moreover, the relationship model between the aggregate model and mechanical properties was successfully established.

In conclusion, the application of an artificial neural network and numerical simulation method for the optimization of device parameters is more conducive to understanding the operating mechanism and achieving rapid optimization of the device. However, the sample data of artificial neural network models do not often derive from rigorous calculation but rather from engineering experience and test measurement. The artificial neural network is mostly used for safety evaluation or prediction. There are relatively few researches on the optimization of device parameters by coupling artificial neural networks with CFD-DEM. The combination of artificial neural network and CFD-DEM coupling not only reduces the errors caused by objective factors but also quickly and accurately predicts the performance of the device under the combination of different parameter solutions. Therefore, in this study, the parameters of the walnut shell-kernel winnowing device were optimized by combining CFD-DEM coupling and an artificial neural network. In order to improve the separation performances of the winnowing device, provide a reference for the optimal design in other fields and to access the efficiency improvement of related enterprises, the influence of inlet wind velocity, baffle opening and inlet angle on separation performance was studied.

## 2 Materials and methods

### 2.1 Design of walnut winnowing device

**FIGURE 1:**
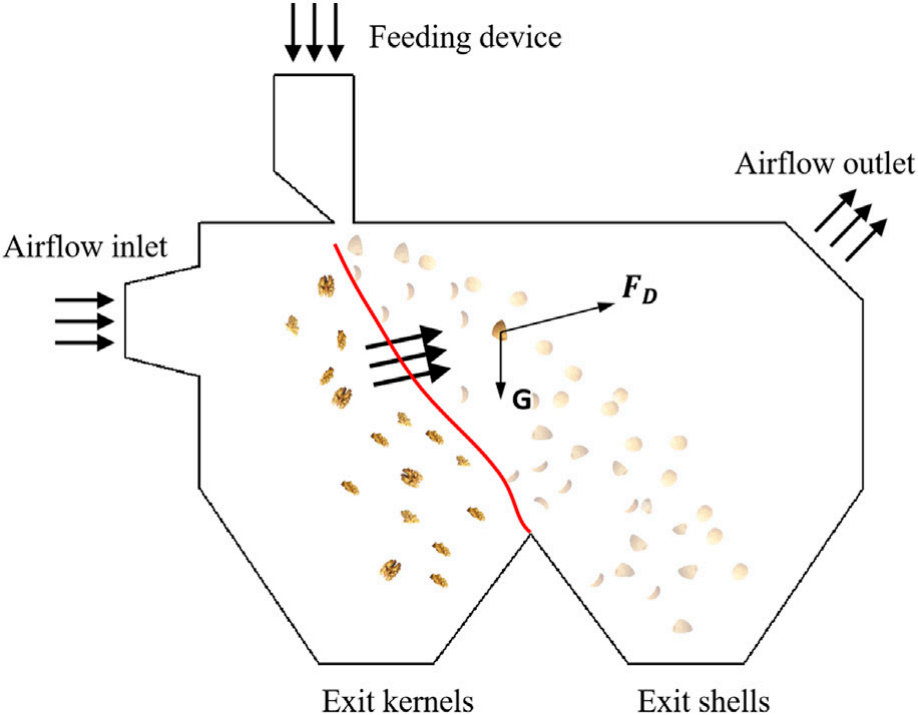
Demonstration diagram of the separation process.

Following shell breaking through air flows, walnut shell-kernel winnowing device can separate walnut shell-kernel mixtures (walnut kernels, walnut shells, Diaphragma juglandis and the crushed materials), yielding pure walnut kernels. The walnut shell-kernel winnowing device is mainly composed of an inlet, outlet, winnowing separation chamber, feeding device and discharge collection device (Figure 1). Following shell breaking, the shell-kernel mixture enters into the winnowing separation chamber through the feeding device and then moves in a quasi-horizontal projectile motion along the resultant force of airflow due to the thrust provided by the draught fan and its gravitational force. Walnut shells and walnut kernels successfully separate from one another in the winnowing separation chamber because of their different mass, density and windward area.

### 2.2 Performance evaluation

#### 2.2.1 Sample preparation

**FIGURE 2:**
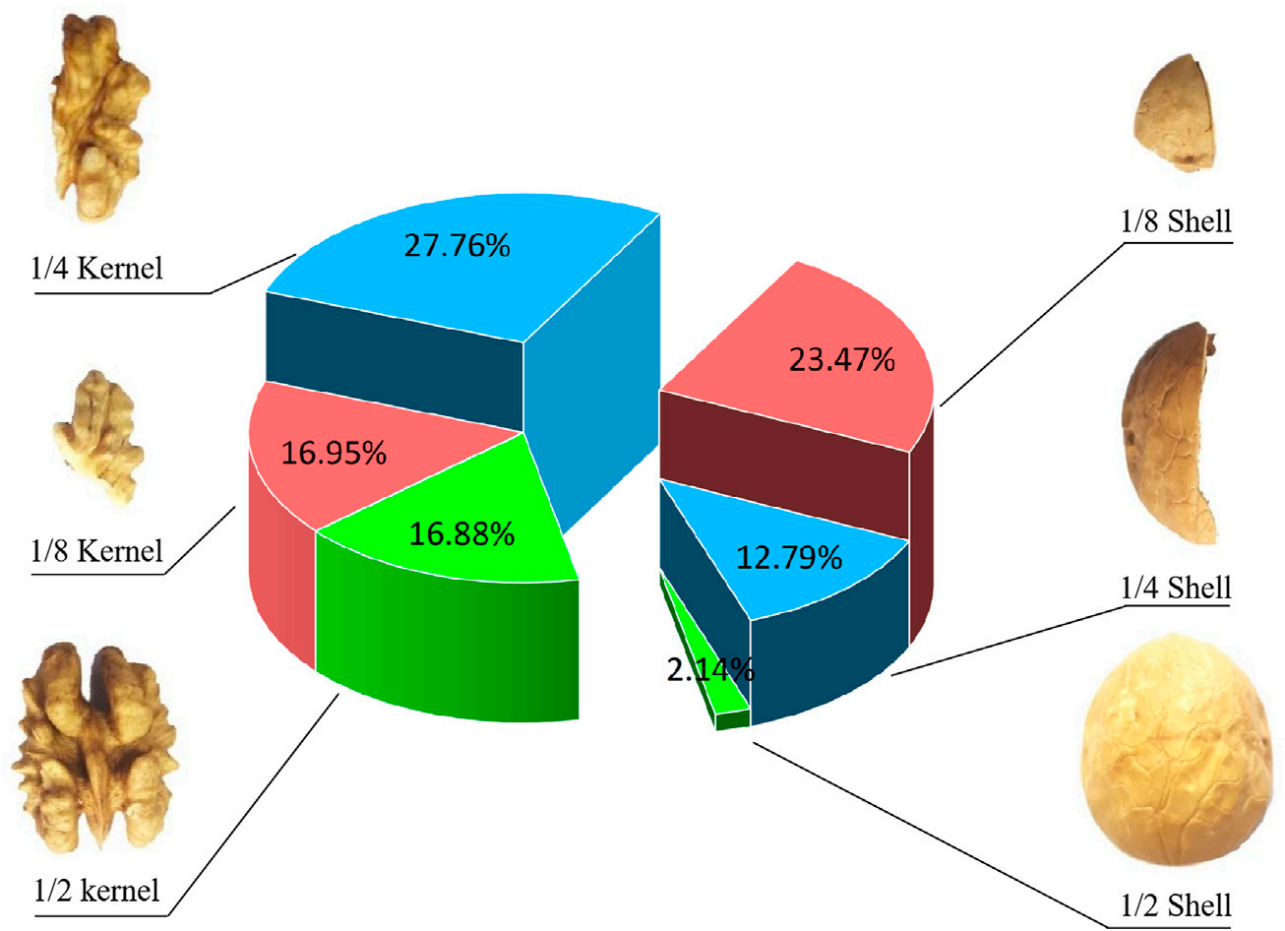
Component proportion diagram of shell-kernel mixtures.

In this study, Wen 185 thin-shell walnut, a major walnut variety in Xinjiang, was used as the sample. The auto-classification squeezing walnut breaking device designed and developed by the research team was used to break walnut samples. The shell-breaking rate was 93.2% and the high kernel rate was 89.8% (Shen et al., 2016). Moreover, the secondary shell breaking was implemented in unbroken walnut shell-kernel mixtures. Five groups of walnut samples (3.00 kg) were collected randomly for shell breaking. The shell-kernel mixtures after shell breaking were collected for screening while obtaining six types of materials including walnut shells (1/2, 1/4, 1/8) and walnut kernels (1/2, 1/4, 1/8). Results are shown in Figure 2. Different groups were separated and weighted followed by the calculation of different material proportions. The mean values were used as the practical proportions.

#### 2.2.2 Performance indexes

To determine the separation performance of the walnut shell-kernel winnowing device, cleaning rate (P, %) and loss rate (Q, %) were used as test indexes. The cleaning rate refers to the degree of thorough material cleaning after separation, as presented in Eq. 1. The loss rate refers to the proportion of walnut kernel loss after material separation, as presented in Eq. 2.

$$\begin{array}{c} P=\frac{m_{w}}{m_{a}}\times 100\% \end{array}$$ (1)

where *P* is the cleaning rate (%), *m*_*w*_ is the walnut kernel mass collected from the outlet (g), and *m*_*a*_ is the sum of all material masses collected from outlet (g).

$$\begin{array}{c} Q=\frac{m_{k0}}{m_{kt}}\times 100\% \end{array}$$ (2)

where *Q* is the loss rate (%), $m_{k0}$ is the walnut kernel mass collected from the outlet (g), and $m_{kt}$ is the sum of all walnut kernel masses in the device (g).

### 2.3 CFD-DEM coupling simulation

Shell-kernel winnowing process is an example of a typical gas-solid flow simulation. The principle behind the accurate description of gas-solid migration and collision lies in whether it is necessary to build an appropriate simulation environment and analyze the gas-solid coupling effect. No matter whether the material-free airflow field numerical analysis or airflow-free particle simulation analysis is used exclusively, research on the winnowing process still has certain limitations (Shi and Sakai, 2022). Therefore, in this paper, EDEM software was used to establish a walnut-crushing material model and Fluent in ANSYS software was used to simulate and analyze the fluid in the air separation chamber. The Fluent module was coupled with EDEM software to simulate the gas-solid coupling two-phase flow of the designed walnut shell human-air separation device. Considering the overly complex structure of the walnut model, the following assumptions were made before simulation to simplify the model:

(1) The airflow in a winnowing separation chamber is defined as an incompressible viscous fluid and the air density as well as the kinematic viscosity are regarded as constants.
(2) The walnut density is assumed to be uniform for the same material.

#### 2.3.1 Construction of discrete element model

Accurate parameter setting and the construction of an accurate particle model are prerequisites for reliable results. Walnut is considered a typical agricultural material. After shell breaking, shell-kernel mixtures could get various shapes, which are difficult to correctly define (Ma et al., 2022). Since the walnut shell-kernel windward area could significantly influence the separation performance, the standard spheres are useful for the description. The irregular walnut fragments were equivalent to standard spheres. The equivalent transformation of the irregular shell-kernel mixtures was implemented based on the spherical diameter of the windward area (Lim et al., 2016).

**FIGURE 3:**
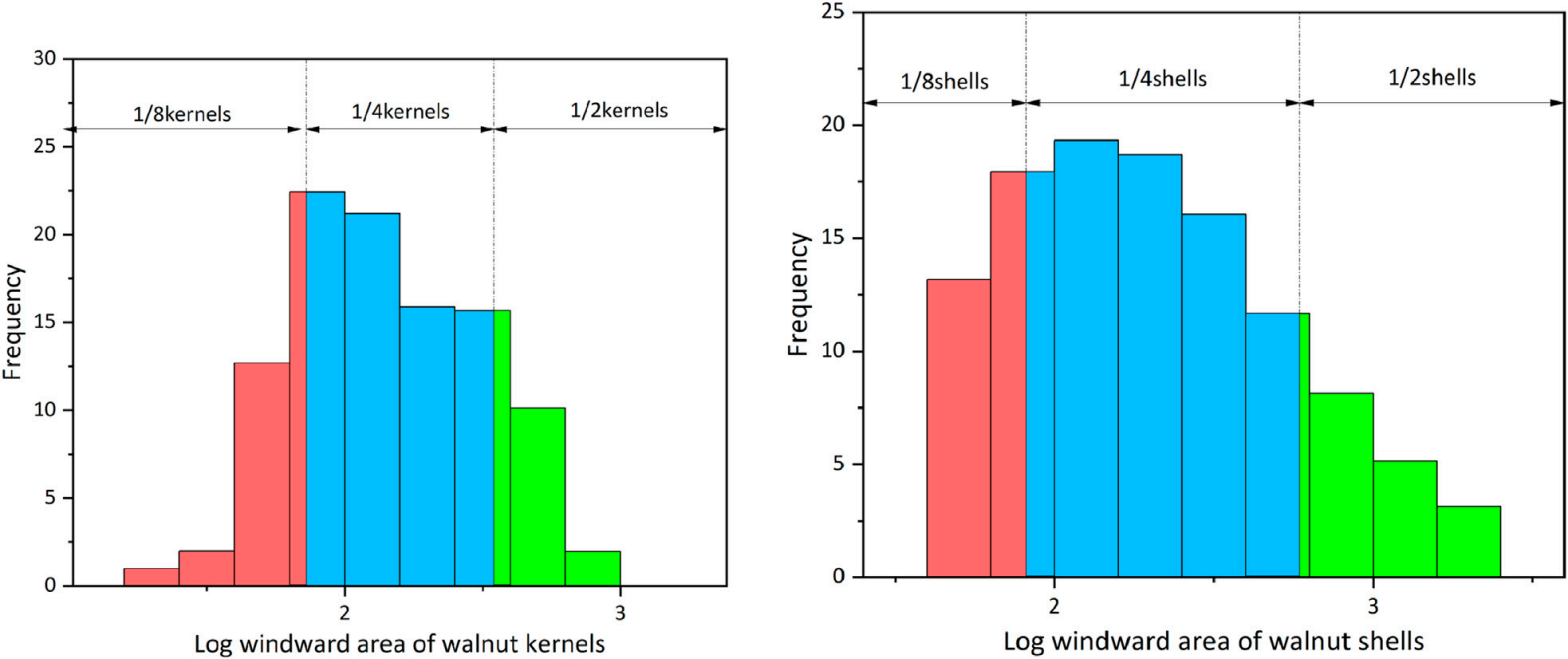
Walnut windward area distributed diagram of numerical frequency. **(A)** Frequency distribution of walnut kernels on the left and **(B)** Frequency distribution of walnut shells on the right.

To determine the windward area distribution of the walnut shell-kernel mixture, the windward areas of shell and kernel samples were tested by image processing. According to statistics, the windward area range of walnut shells was 6.15–1085.56 mm^2^ while the windward area range of walnut kernels was 5.18–827.15 mm^2^ (Figure 3).

As shown in Figure 3, both shell and kernel windward areas followed the logarithmic normal distribution. To select representative parameter values with statistical significance, the logarithmic normal distribution was divided into three regions by using the “3 Sigma criterion” (Simone et al., 2017). The distribution values in the 1 and 2 standard deviation ranges were about 68% (blue part in Figure 3A) and 95% (red, blue and green parts in Figure 3B), respectively. According to the three split parts, volume percentages (trapezoidal numerical integral) of walnut shells and kernels were calculated. Finally, the weighted mean of particles in each size section was used to represent the logarithmic value of the particle windward area. It was substituted into the calculation formula (Eq. 4) of equivalent spherical diameter and the equivalent spherical diameters were obtained (Table 1). The required particle density was calculated according to Eq. 5 based on the equivalent spherical diameter of the equivalent windward area.

$$\begin{array}{c} \varphi_{i}=\frac{\sum_{j}S_{ij}∙p_{ij}}{\sum_{j}p_{ij}} \end{array}$$ (3)

$$\begin{array}{c} d_{s}=2\sqrt{\frac{\mathrm{lg}\varphi_{i}}{\pi }} \end{array}$$ (4)

$$\begin{array}{c} \rho =\frac{\pi d_{s}^{3}}{6} \end{array}$$ (5)

where *i* represents the segment number and j represents the points contained in this part segment; $S_{ij}$ is the j-size class and $p_{ij}$ is the volume probability of each class *j*. Regions of different categories are expressed by different colors in Figure 3, where $d_{s}$ is the equivalent spherical diameter of irregular shells and kernels (mm) and $\varphi_{i}$ is the equivalent windward area of irregular shells and kernels (mm^2^).

**TABLE 1:** Windward area equivalent ball diameter information table.

Component (walnut shells)	$d_{s}/\left(mm\right)$	Component (walnut kernels)	$d_{s}/\left(mm\right)$
1/2 shells	4.19	1/2 kernels	4.63
1/4 shells	6.77	1/4 kernels	8.14
1/8 shells	12.42	1/8 kernels	16.59

#### 2.3.2 Construction of computational fluid dynamics (CFD) and model verification

**FIGURE 4:**
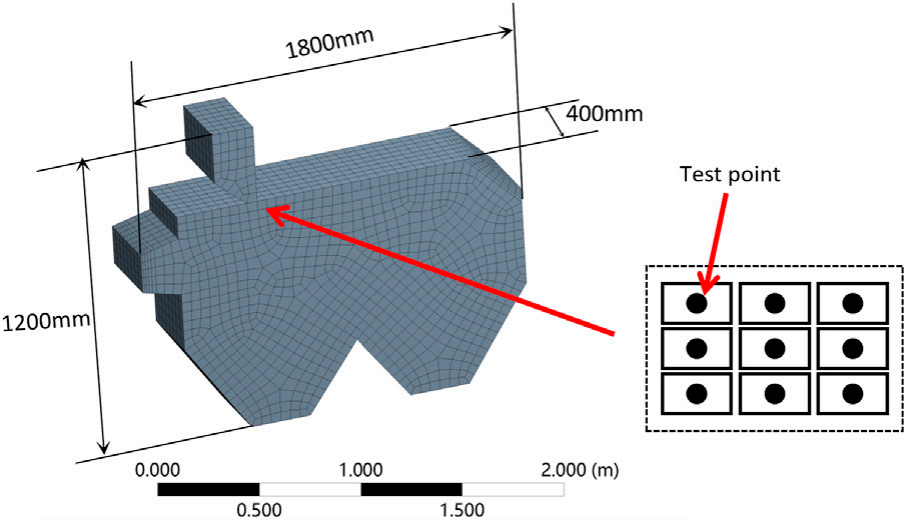
Winnowing device room size labeling and grid diagram.

Even though limited by the computational capacity of servers and the simulation ability of the software, the winnowing device was simplified appropriately and then a model was built using professional modeling software (Figure 4).

Boundary conditions are essential to calculate the motion boundaries of an airflow. The airflow inlet was established as the velocity inlet. The inlet turbulent flow was defined by 5% turbulence intensity and the hydraulic diameter of 218.18 mm. The outlet boundary was established as the pressure outlet. The outlet turbulent flow was defined by 5% turbulence intensity and the hydraulic diameter of 342.85 mm. The surface boundary was established as the no-slip boundary. The heat transfer among winnowing airflows, walnut shell-kernel and wall surface was ignored.

**FIGURE 5:**
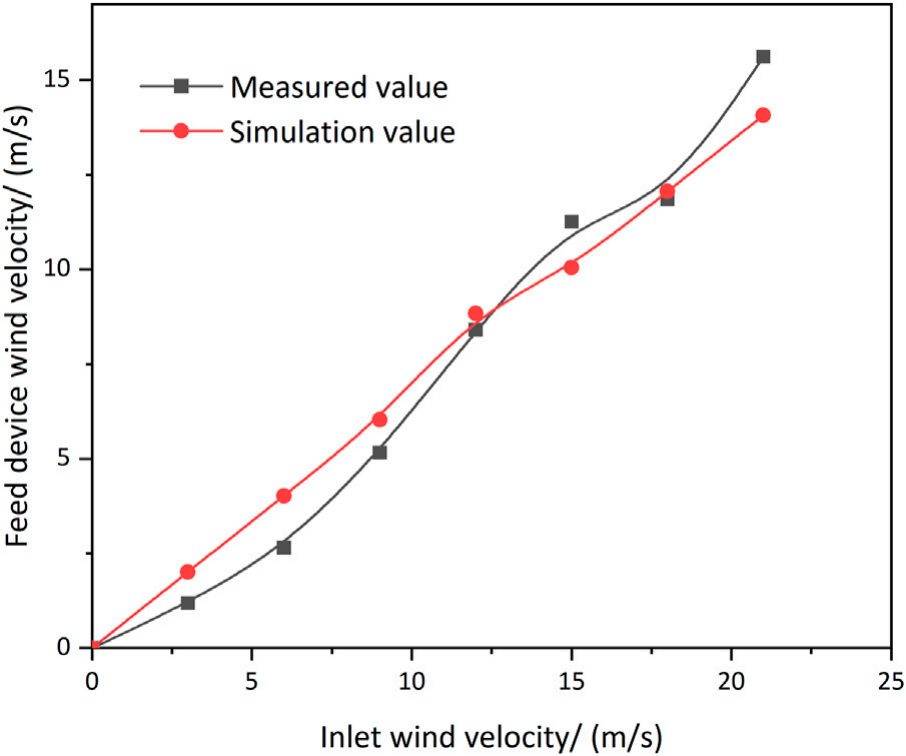
Comparison of measured and simulated values.

In order to verify the accuracy and reliability of the calculation on the established winnowing separation chamber model, nine-point sampling was carried out at the feeding device (Figure 4) and the measured wind speed of each measuring point at the feeding device was compared with the simulated value (Figure 5). To obtain the measured value, the induction probe of the thermal anemometer was placed on nine measuring points of the same measuring section. The average wind speed of nine measuring points was considered at this place to complete the single wind speed measurement. The simulation value acquisition and EnSight module on the ANSYS platform were used to extract wind speed based on nine-point sampling.

As shown in Figure 5, there was a certain deviation between the measured value and the simulated value of wind speed. However, the overall distribution and variation trend were consistent. It demonstrated that the model established in this simulation met the simulation requirements. The meshing was moderate and the boundary conditions were found to be reasonable. It can be concluded that the established CFD model is effective and can be used for the follow-up test.

#### 2.3.3 DDPM coupling parameter setting

The grid model was input into DEM. Property parameters and collision contact parameters of walnut shells and kernels were established. According to the proportion of different types of shells and kernels after shell breaking (Section 2.2.1), the ratios of simulation particles were established. The Hertz-Mindlin (no-slip) contact model was utilized in the dense discrete phase model (DDPM) model, which was based on the calculation of particle volume fraction. The gravitational acceleration was established according to the practical gravity direction, a comparatively accurate Realizable k-ε turbulence model was chosen to calculate the fluid domain. The time step length of DEM was chosen to be 20%–30% of Rayleigh’s time step in order to balance accuracy and calculation time. Time step length was established as 1 × 10^−8^ s and the Fluent calculation time step length was established as 1 × 10^−6^ s.

### 2.4 Experimental design

According to relevant literatures and pre-tests in early stage (Yuan et al., 2018), it decides to optimize technological parameters of the separation device first under the premise of successful walnut shell-kernel winnowing, including opening of baffle, inlet wind velocity and angle of inlet. The statistics data of the artificial neural network training set and verification set was determined using the central composite design (CCD) test. The factor level coding was decided and presented in Table 2.

**TABLE 2:** Test factors horizontal coding table.

Levels	Opening of baffle/(cm)	Inlet wind velocity/(m/s)	Angle of inlet/(°)
−1	6	22	8
0	7	24	10
1	8	26	12

All experiments had three parallel tests and the results were expressed by mean values. Optimization data analysis was carried out using the neural network platform of JMP Pro.

## 3 Results and analysis

### 3.1 Experimental design and construction of a neural network model

Based on the principle of CCD test design, orthogonal factor and repetition times at the center point were established as 1.633 and 6, respectively. The experimental design and results are shown in Table 3.

**TABLE 3:** Test design matrix and test results.

Num	Model	Opening of baffle/(cm)	Inlet wind velocity/(m/s)	Angle of inlet/(°)	Loss rate/(%)	Cleaning rate/(%)
1	++−	8	26	8	24.57 ± 3.12	83.92 ± 5.34
2	−++	6	26	12	22.76 ± 4.89	77.60 ± 2.11
3	−−−	6	22	8	16.00 ± 4.01	65.93 ± 2.36
4	000	7	24	10	14.86 ± 2.09	77.15 ± 3.14
5	000	7	24	10	12.25 ± 3.49	76.13 ± 2.65
6	+−+	8	22	12	21.74 ± 6.45	66.74 ± 5.63
7	000	7	24	10	15.78 ± 4.62	81.34 ± 3.18
8	−+−	6	26	8	19.91 ± 1.99	75.39 ± 5.24
9	+−−	8	22	8	22.00 ± 2.51	73.53 ± 3.15
10	000	7	24	10	15.40 ± 3.89	78.23 ± 4.68
11	+++	8	26	12	21.06 ± 3.14	74.03 ± 5.23
12	−−+	6	22	12	22.00 ± 2.58	68.37 ± 1.85
13	00a	7	24	6.7340	14.50 ± 2.36	71.95 ± 3.46
14	000	7	24	10	15.00 ± 2.38	83.14 ± 2.53
15	00A	7	24	13.2650	17.30 ± 4.36	72.23 ± 3.16
16	A00	8.6330	24	10	20.76 ± 3.98	75.32 ± 2.56
17	0a0	7	20.73401	10	23.26 ± 4.11	63.70 ± 3.27
18	000	7	24	10	11.73 ± 5.12	79.34 ± 4.36
19	a00	5.3670	24	10	16.93 ± 3.25	71.07 ± 4.12
20	0A0	7	27.2660	10	25.82 ± 4.36	75.80 ± 2.16

The above orthogonal test results were used as the training set and verification set. A neural network model was constructed using JMP Pro14.0 (SAS) platform. The samples were divided into K groups (K folds, where K equal to 5) randomly without repetitions by choosing the “K-fold” crossing verification method. Among them, 1 group was used for verification and the rest (K-1) groups were used for training. The separation performances were optimized.

Through multiple analyses of the training set and verification set, a 3-input and 2-output neural network model was determined. The 3 input neurons represented the baffle opening, inlet wind velocity and inlet angle. There were nine neurons in the two hidden layers. The 2 output neurons represented the cleaning rate (P) and loss rate (Q) of separation performances, respectively. The hidden layer structure was established. The S-shaped TanH function, the identical linear function and the radial Gaussian function were activated to increase the learning rate to 0.1. The transformation covariable was chosen as a fitting option and robust fitting was chosen to prevent overfitting. The penalty method used “square.” The fitting process of this neural network model was executed while obtaining R^2^ of the loss rate (96.37%) and the cleaning rate (89.72%).

### 3.2 Optimization and analysis of neural network model

**FIGURE 6:**
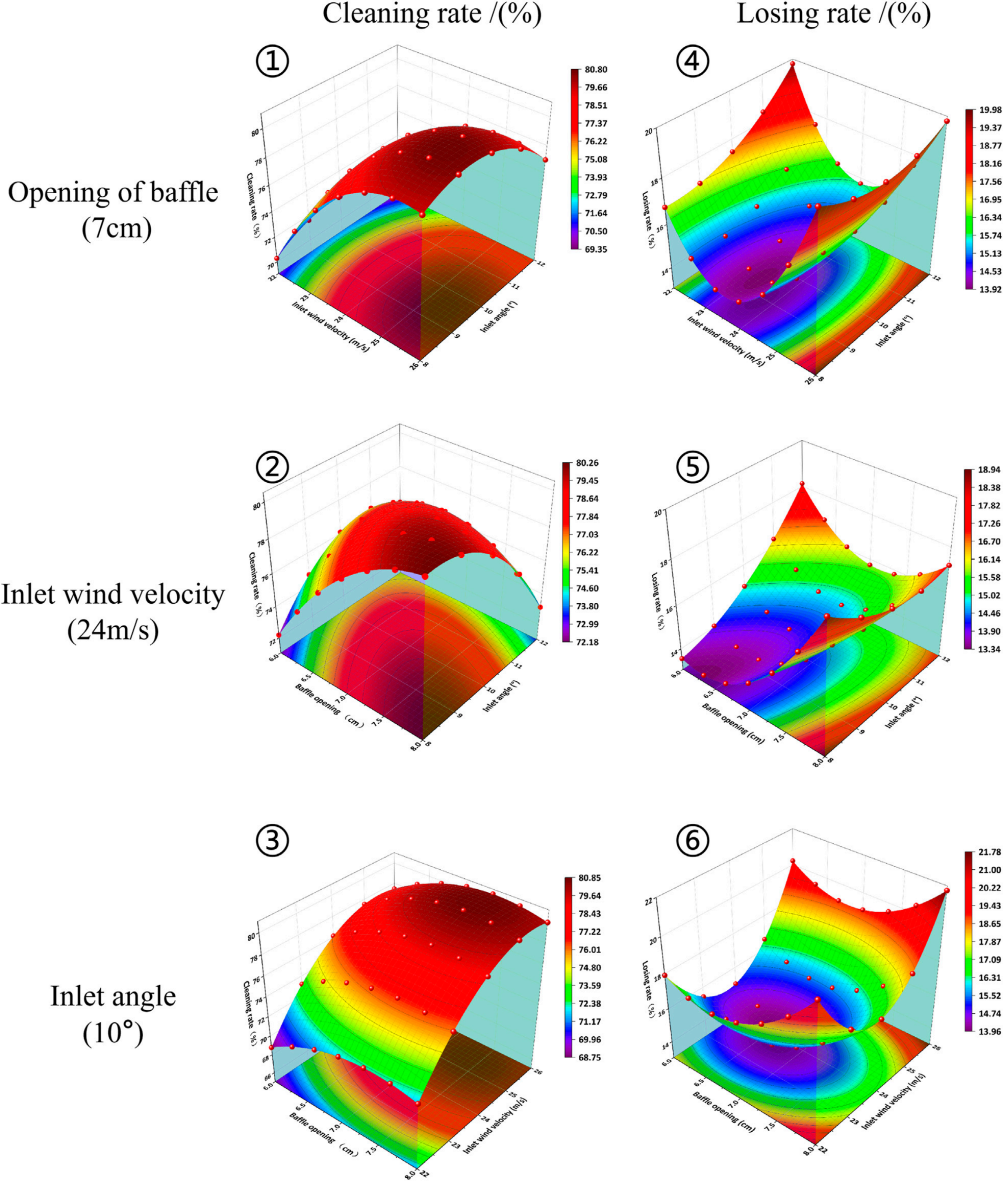
The effects of various factors on separation performance.

The neural network prediction model produced the prediction formula and the three-dimensional response curved surface diagram was plotted (Figure 6). The red part in the figure represents high level and the blue part represents the low level. As shown in Figure 6-①, when the baffle opening was 7 cm, the cleaning rate increased gradually with the increase in inlet wind velocity. Meanwhile, the minimum cleaning rate was achieved when the inlet angle was fixed and the inlet wind velocity was 22 m/s. The maximum cleaning rate was achieved when the wind velocity was 25.5 m/s. As shown in Figure 6-②, the maximum cleaning rate was achieved when the inlet wind velocity was 24 m/s, the baffle opening was 7.75 cm and the inlet angle was 8.50°. With the reduction in baffle opening and the increase in inlet angle, the cleaning rate declined. As shown in Figure 6-③, the cleaning rate increased with the increase in inlet wind velocity while having a constant inlet angle at 10°. However, it started to decline when the inlet wind velocity approached 26 m/s. Based on the above results, inlet wind velocity was found to significantly influence the cleaning rate while baffle opening and inlet angle had a minor influence.

According to Figure 6-④, the loss rate first decreased and then increased with the increase in inlet wind velocity while having a 7 cm baffle opening. The optimal loss rate was obtained when the inlet wind velocity was 23.5 m/s. The loss rate was higher than 16% when inlet wind velocity was 22 m/s or 26 m/s. As shown in Figure 6-⑤, baffle opening and inlet angle was positively related to loss rate when the inlet wind velocity was 24 m/s. In other words, loss rate increased with the increase in baffle opening and inlet angle. As shown in Figure 6-⑥, when the inlet angle was 10°, loss rate first decreased and then increased with the increase in baffle opening and inlet wind velocity. The inlet wind velocity had a relatively significant influence while the baffle opening had a minor influence. To sum up, inlet wind velocity was the primary influencing factor of loss rate, followed by inlet angle and baffle opening.

**FIGURE 7:**
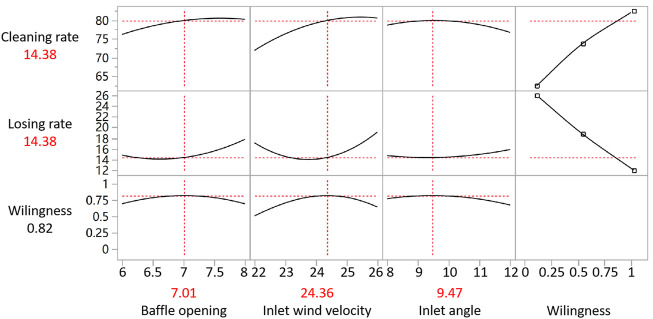
The trend chart of prediction descriptor.

The prediction descriptor plotted by the JMP software is shown in Figure 7. Within the given range of factor conditions, the cleaning rate increased with the increase in baffle opening and inlet wind velocity, while the cleaning rate declined with the increase in inlet angle. In the given range of reaction conditions, the maximum cleaning rate was achieved when the baffle opening was 8 cm, the inlet wind velocity was 25.49 m/s and the inlet angle was 8.18°. At this moment, the cleaning rate was kept at 81.91%. The verification test was carried out under the maximum willingness. The cleaning rate was determined to be 82.16%, which agreed with the software fitting outcomes. Compared to the cleaning rate, the maximum willingness value of loss rate was achieved when the baffle opening was 6.28 cm, the inlet wind velocity was 23.54 m/s and the inlet angle was 8.01°. Under this circumstance, the cleaning rate stabilized at 13.14%. Based on the combined prediction of the cleaning rate and loss rate, the cleaning rate was maintained at 79.91% and the loss rate was stabilized at 14.38% while having the baffle opening of 7.01 cm, inlet wind velocity of 24.36 m/s and inlet angle of 9.47°. On this basis, the optimal prediction parameter combination was acquired. For the optimal parameter combination, the cleaning rate, product quality and value improved while having a normal operation. Meanwhile, the low loss rate reduces the optimization caused by the improper selection of products and avoids the waste of resources, which is the result of neural network optimization. The above results were obtained based on the neural network prediction model which was required to be verified by an experiment.

### 3.3 Accuracy test of neural network model

To test the reliability of the neural network model, simulation tests based on five groups of new factor-level combinations were carried out. Results were compared with the prediction results of neural network to determine the accuracy and stability of the artificial neural network predictions (Table 4).

**TABLE 4:** Test factors horizontal coding table.

	Factors			Cleaning rate/(%)			Loss rate/(%)		
Groups	Opening of baffle/(cm)	Inlet wind velocity/(m/s)	Angle of inlet/(°)	Predicted values	Simulation values	Relative error (%)	Predicted values	Simulation values	Relative error (%)
1	6	24	8	72.18	70.22	2.72	13.52	12.59	6.88
2	7	26	10	80.35	79.69	0.82	19.06	20.14	5.67
3	8	22	10	71.70	72.97	1.77	20.85	22.76	9.16
4	7	24	12	69.37	70.17	1.15	19.80	21.05	6.31
5	8	26	12	73.81	72.99	1.11	20.95	22.58	7.78

The prediction values of the neural network model and simulation results are presented in Table 4. The relative error range of the cleaning rate was 0.82%–2.72% and the relative error range of the loss rate was 5.67%–9.16%, indicating that both the cleaning rate and loss rate were kept within a small error range. The constructed neural network model demonstrated a good prediction ability and can be used for separation performance prediction analysis of the winnowing device.

### 3.4 CFD-DEM coupling simulation analysis

**FIGURE 8:**
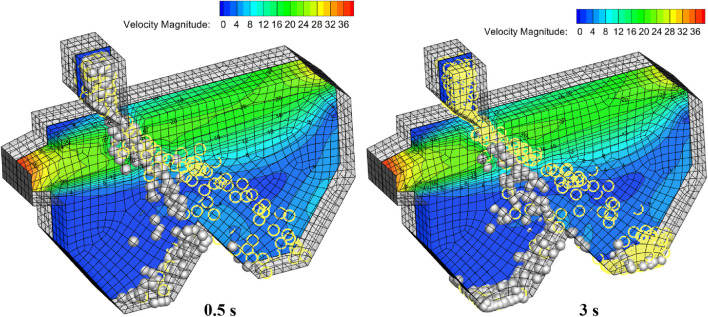
Material particles position transient map.

The transient diagrams of the winnowing device’s airflow field at 0.5 and 2 s are shown in Figure 8. Statistical zones of loss rate and cleaning rate were established at the shell outlet and kernel outlet, respectively. After falling into the action zone of airflow, the shell and kernel developed different motion tracks under the inclined airflows.

#### 3.4.1 CFD-DEM coupling practical simulation effect analysis

**FIGURE 9:**
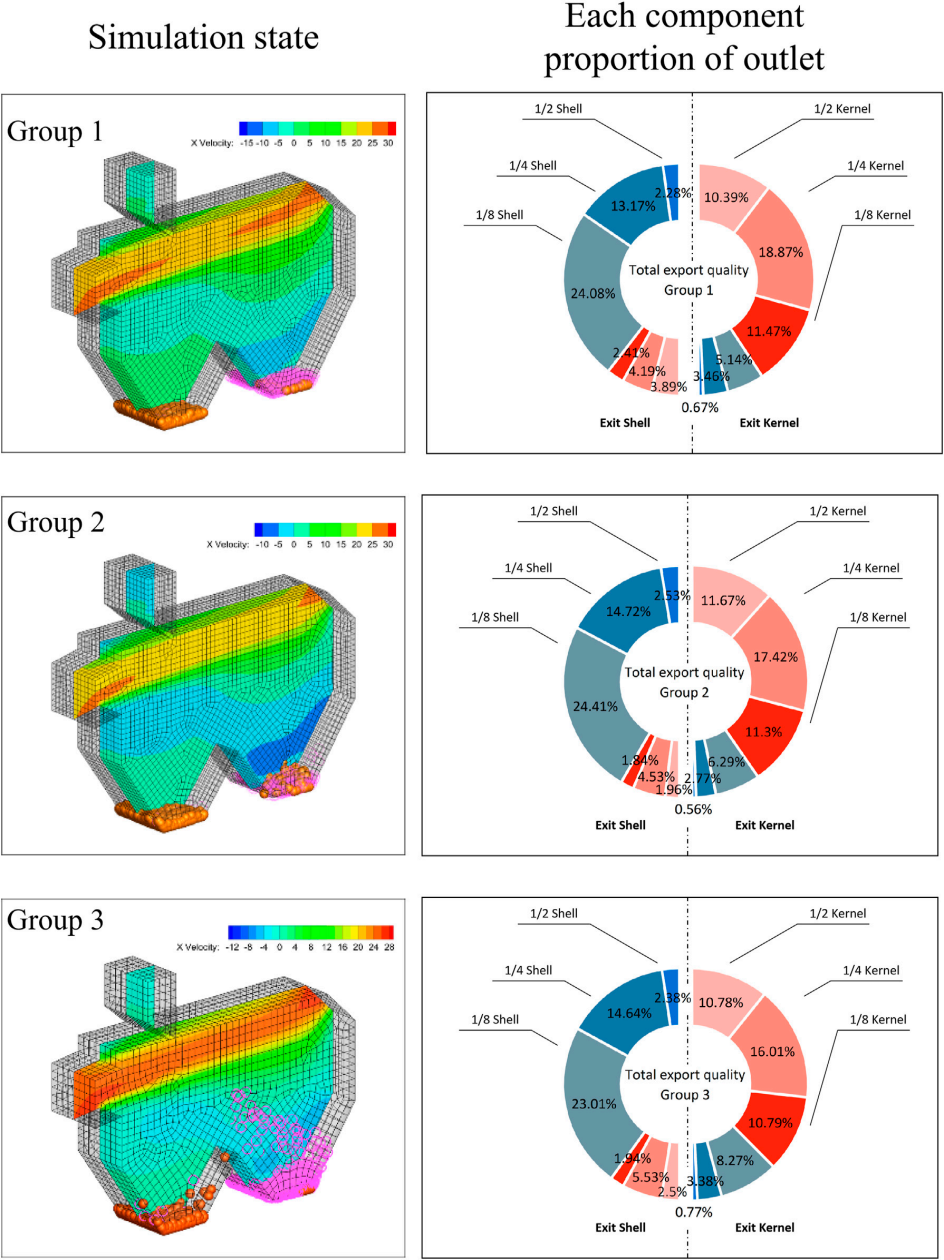
Simulation status and proportion chart.

The CFD-DEM coupling practical separation effect is shown in Figure 9. The best separation performance was achieved by Group 2, which was therefore considered the optimal parameter group. Data from different groups are presented in Table 5. Practical simulation separation performance and prediction results differed slightly. The separation performance of Group 1 was better than that of Group 2. Its cleaning rate and loss rate increased by 1.17% and 8.15%, respectively. However, the comprehensive separation performance declined. Compared to Group 1, the cleaning rate of Group 3 decreased by 5.23% and the loss rate increased by 2.99%. The separation performance of Group 3 was poor.

**TABLE 5:** Comparison test group.

	Factors			Cleaning rate/(%)			Loss rate/(%)		
Groups	Opening of baffle/(cm)	Inlet wind velocity/(m/s)	Angle of inlet/(°)	Predicted values	Simulation values	Relative error (%)	Predicted values	Simulation values	Relative error (%)
1	8.01	25.36	7.47	81.62	82.89	1.56	22.53	23.69	5.15
2	7.01	24.36	9.47	79.91	78.11	2.23	14.38	13.78	4.17
3	6.01	23.36	11.47	74.68	72.49	2.93	17.37	18.02	3.74

As shown in Figure 9, the left side represents the simulation state and the right side represents the mass proportion of each component collected at the exit shell and exit kernel. Moreover, walnut kernels doped with a lot of impurities were collected at the kernel outlet of Group 3. The cleaning rate was found to be relatively poor. Many wrongly classified walnut kernels were collected at the shell outlet, which increased the loss rate. As shown in Figure 9, materials collected at all outlets in each test were used as the general data.

Compared to test Group 2, the analysis of the proportion of each component in the export of 1/2, 1/4, and 1/8 walnut shells in test Group 3 showed that the walnut shells were increased by 0.21%, 0.61%, and 1.98%. Moreover, 1/8 walnut shells were improperly selected, which resulted in the poor cleaning rate of the winnowing device. As shown in Group 2 Figure 9, walnut kernels collected at shell outlet decreased. The 1/2, 1/4, and 1/8 walnut kernels were decreased by 0.10%, 1.00%, and 0.54%, respectively, while many 1/4 walnut kernels were blown to the shell outlet. With the increase in inlet wind velocity, a higher number of 1/8 walnut shells and 1/4 walnut kernels are wrongly classified, which decreased the cleaning rate and increased the loss rate accordingly.

Similarly, relatively pure walnut kernels were collected at the kernel outlet of Group 1, indicating a relatively high cleaning rate. Moreover, a higher amount of walnut kernels were collected at the shell outlet, resulting in a high loss rate. Since the baffle opening and inlet wind velocity were at a high level, the flight coefficients of the shell and kernel were relatively high. It resulted in a relatively high cleaning rate. Nevertheless, inlet wind velocity and inlet angle were both beneficial for airflow to act on walnut fragments better. As the difference in flight coefficients was relatively small, it resulted in a high loss rate. In this section, it was explained by the flight coefficient theory. However, shell-kernel mixtures demonstrated different shapes after breaking with varied influencing factors. Besides, since the suspending speeds of walnut shells and kernels were partially overlapped, the winnowing technique failed to complete shell-kernel separation. This is the next problem that requires urgent attention.

#### 3.4.2 Verification test

**FIGURE 10:**
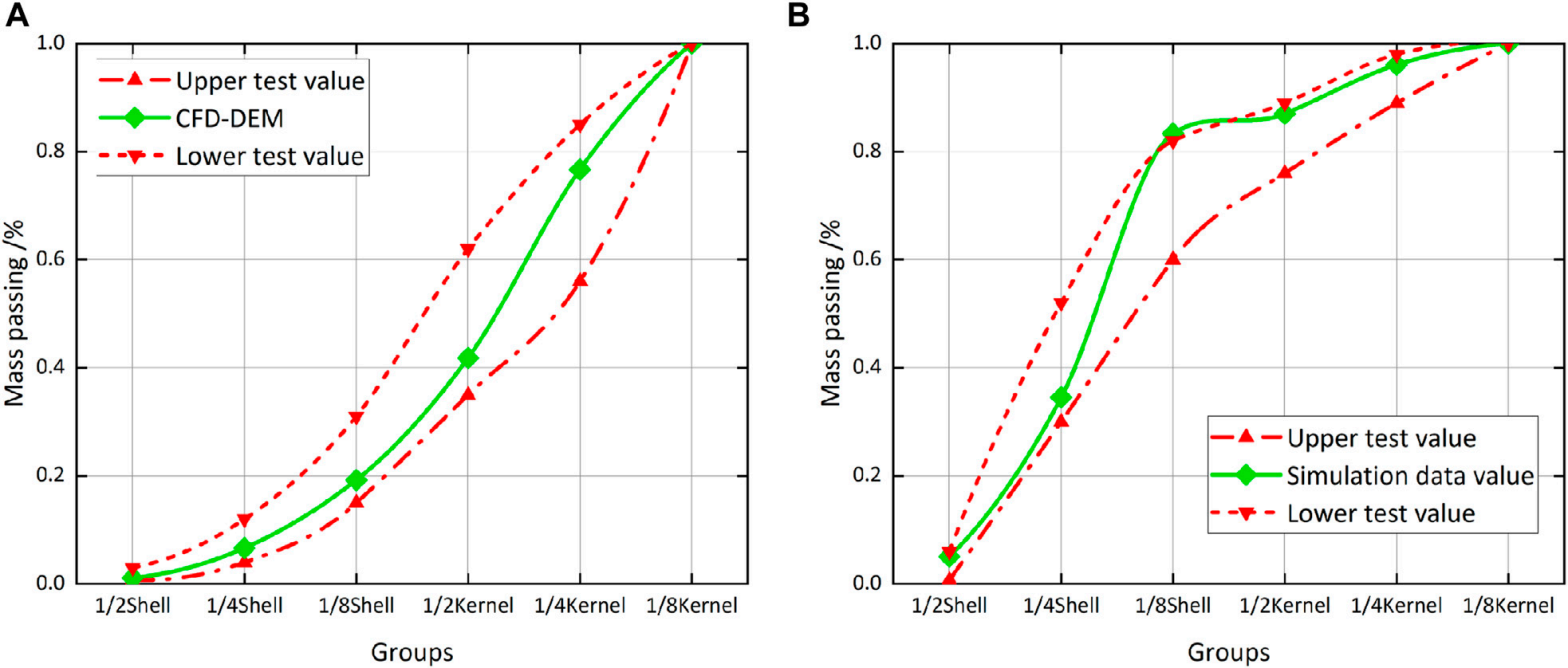
Simulation status and proportion chart. **(A)** Each material quality at the kernel outlet passes through **(B)** Each material quality at the shell outlet passes through.

A bench verification test was carried out to determine the reliability of the prediction model and simulation test. The shell-kernel mixture was placed into the tray for later use. The power was then supplied to start the draught fan. Wind speed at the inlet was measured by an anemoscope and the speed switch was adjusted to get the required wind during the test. After the wind speed of the draught fan was stabilized, the shell-kernel mixed particles in the tray were discharged from the feeding mouth of the winnowing separation chamber. The baffle was fixed after being adjusted to the predicted opening. Samples from the kernel outlet and shell outlet were collected and weighed once the test was complete. The cleaning rate and loss rate of walnut kernels in the winnowing device were calculated (Figure 10).

Ten tests were performed to verify the reliability of the model. A statistical analysis of the upper and lower limits of cleaning rate and loss rate was carried out, which was marked by the red dotted line in Figure 10. The dotted line represented the upper and lower limits of multiple test results. If the walnut simulation data (green solid line) is within the dotted line range, the simulation agrees with practical data. As shown in Figure 10A, green solid lines were all kept within two green dotted lines, indicating that simulation test data at the kernel outlet had relatively high reliability. As shown in Figure 10B, most green solid lines were within two red dotted lines, indicating that the simulation test was highly consistent with test results. However, a lot of 1/8 shells were collected at the shell outlet. Although it exceeded the upper limit of the test (1.06%), it was still in the acceptable range.

## 4 Conclusion

The simulation model of the device and its research object can be swiftly established by using the CFD-DEM coupling. The gas-solid two-phase flow and the interaction between them can be well simulated. However, the application of the CFD-DEM coupling in the industrial and agricultural fields is still in the initial stage and hence, its application potential needs to be further investigated. For example, to improve the accuracy and reliability of the model, the contact model and the model parameters need to be accurately selected and established. The universality and authenticity of the coupling simulation method and the computational efficiency of the model are required to be further improved. At the same time, neural networks and other advanced technical means must be integrated with numerical simulation to eliminate the shortcomings of numerical simulation. Considering the large demand for computing power and the large number of experiments, it will become a trend in the future development of numerical simulation. The main conclusions of this paper are as follows:

(1) In this study, an artificial neural network prediction model of a walnut shell-kernel winnowing device was constructed. The baffle opening, inlet wind velocity and inlet angle were used as the input factors while the cleaning rate and loss rate were used as output indexes. Results demonstrated that the average relative error of the cleaning rate of the model was 1.77% and R^2^ was 89.72%. The average relative error of loss rate was 7.42% and R^2^ was 96.37%. This proves that this prediction model has good prediction capability.
(2) The operation mechanisms of the walnut shell-kernel winnowing device under different parameter combinations were investigated. According to response surface analysis, inlet wind velocity was the primary influencing factor of the device’s cleaning rate, followed by baffle opening and inlet angle. Moreover, inlet wind velocity was the primary influencing factor of loss rate, followed by inlet angle and baffle opening. Meanwhile, a higher number of 1/8 walnut shells and 1/4 walnut kernels were misclassified with the increase in inlet wind velocity, which deteriorated the device’s performance.
(3) The prediction descriptor plotted by the artificial neural network prediction model was analyzed while obtaining the optimal parameter combination for the walnut shell-kernel winnowing device with baffle opening = 7.01 cm, inlet wind velocity = 24.36 m/s and inlet angle = 9.47°. Under this optimal parameter combination, the highest cleaning rate (79.91%) under the low loss rate (14.38%) can be achieved while obtaining the optimal winnowing effect of the device.

## Data Availability

The original contributions presented in the study are included in the article/supplementary material, further inquiries can be directed to the corresponding author.
